# The Associated Factors of Low Birthweight Among Term Singletons in Japan: A Pregnancy Birth Registry Analysis

**DOI:** 10.2188/jea.JE20210483

**Published:** 2023-09-05

**Authors:** Yoshifumi Kasuga, Miho Iida, Yuya Tanaka, Masumi Tamagawa, Keita Hasegawa, Satoru Ikenoue, Yasunori Sato, Mamoru Tanaka, Daigo Ochiai

**Affiliations:** 1 Department of Obstetrics and Gynecology, Keio University School of Medicine, Tokyo, Japan; 2 Department of Preventive Medicine and Public Health, Keio University School of Medicine, Tokyo, Japan

**Keywords:** low birthweight, term birth, small for gestational age, underweight, gestational weight gain

## Abstract

**Background:**

Progress in reducing the global low birthweight (LBW) has been insufficient. Although the focus has been on preventing preterm birth, evidence regarding LBW in term births is limited. Despite its low preterm birth prevalence, Japan has a higher LBW proportion than other developed countries. This study aimed to examine the prevalence of LBW in term singleton births and its associated factors using a national database.

**Methods:**

We retrospectively analyzed the data of neonates registered in the Japan Society of Obstetrics and Gynecology Successive Pregnancy Birth Registry System who were born 2013–2017. Exclusion criteria included stillbirths, delivery after 42 gestational weeks, and missing data. Logistic regression analyses were performed to investigate the maternal and perinatal factors associated with LBW in term singletons using the data of 715,414 singleton neonates.

**Results:**

The overall prevalence of LBW was 18.3%, and 35.7% of LBWs originated from singleton term pregnancies. Multiple logistic regression analyses indicated that both modifiable and non-modifiable factors were independently associated with LBW in term neonates. The modifiable maternal factors included pre-pregnancy underweight, inadequate gestational weight gain, and smoking during pregnancy, while the non-modifiable factors included younger maternal age, nulliparity, hypertensive disorders of pregnancy, cesarean section delivery, female offspring, and congenital anomalies.

**Conclusion:**

Using the Japanese pregnancy birth registry data, more than one-third of LBWs were found to originate from singleton term pregnancies. Both modifiable and non-modifiable factors were independently associated with LBW in term neonates. Prevention strategies on modifiable risk factor control will be effective in reducing LBW worldwide.

## INTRODUCTION

Low birthweight (LBW) is defined as a birthweight less than 2,500 g. LBW neonates not only have a higher risk of infant mortality but are also susceptible to long-term health consequences, such as a lower intelligence quotient^1^ and a higher risk of noncommunicable diseases (NCD) in the future.^2^ Reducing LBW has long been recognized as a public health priority; however, in 2015, 20.5 million neonates were born with LBW worldwide, corresponding to a prevalence of 14.6%.^3^ Given that nearly half of LBW neonates born globally are reported in Southern Asia, Asians are one of the most vulnerable populations for LBW and, thus, for future health disorders.^3^ Although lower-middle- and low-income countries have achieved a large reduction through integrated efforts, it continues to be a public health priority, especially in upper-middle and high-income countries, where the decrease is slower.^4^

To design effective strategies for reducing LBW, it is important to investigate a wide range of associated factors, including maternal and perinatal factors. Smoking, alcohol, drugs, perinatal complications like hypertensive disorders of pregnancy (HDP), pre-pregnancy underweight (body mass index [BMI] <18.5 kg/m^2^), inadequate gestational weight gain (GWG), and educational background have been shown to be associated with increased LBW risk.^5^^–^^7^ However, most studies to date have focused on preterm infants, and few studies have clarified these associations in neonates born at term. In our previous single-institutional study of 1,028 LBW neonates, 27.1% were singletons delivered at or after 37 gestational weeks, and 12.6% of multiple births were born at term.^8^ Other reports also suggest that Japan has one of the lowest proportions of preterm births but has a higher prevalence of LBW compared with other developed countries (eFigure 1).^4^^,^^9^^–^^13^ Because factors associated with LBW may differ among different groups, clarification of these associations is crucial in term singleton births; however, there is a lack of available data on the prevalence of LBW and the factors potentially associated with LBW among term neonates.

Therefore, we conducted a retrospective study using a national perinatal database to examine the factors associated with LBW among term singleton births in Japan. We aimed to clarify the factors that independently contribute to LBW in singleton term pregnancies, thereby contributing to future strategic planning in reducing LBW.

## METHODS

### Study design and participants

**Figure 1.**
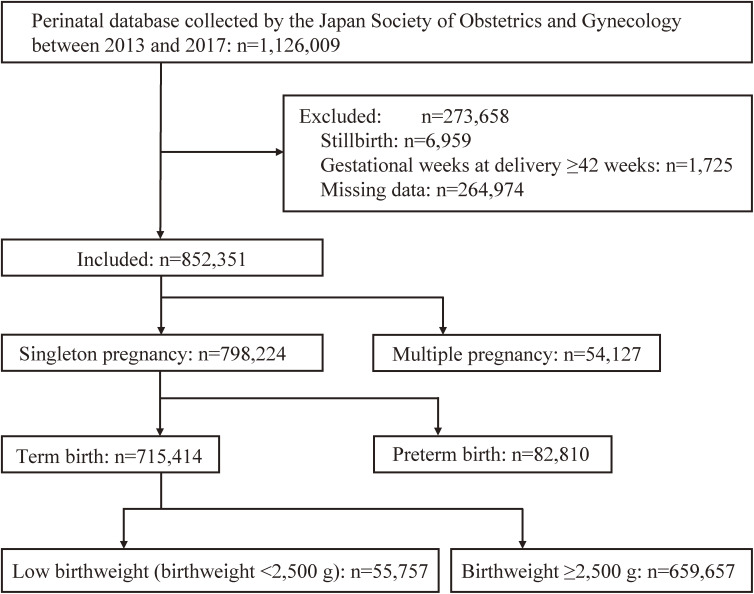
Flow diagram defining study population and exclusions.

The present study was a retrospective investigation of infants whose perinatal data were registered in the Japan Society of Obstetrics and Gynecology Successive Pregnancy Birth Registry System, which has been described elsewhere.^14^ Briefly, it is an ongoing national registry consisting of approximately 400 secondary and tertiary hospitals nationwide. The database collects information on maternal demographics, pregnancy complications, and delivery outcomes extracted from medical records of each institution using a standardized format to understand various issues that need to be resolved at the national level. A total of 1,126,009 births were registered in the system between January 1, 2013, and December 31, 2017. After excluding stillbirths (*n* = 6,959), gestational age at delivery ≥42 weeks (*n* = 1,725), and missing data (*n* = 264,974), 852,351 neonates were selected. Missing data included the lack of data on maternal age, maternal weight (pre-pregnancy or at delivery), maternal height, neonate’s sex, birthweight, and gestational age at delivery. There were 54,127 multiple births and 798,224 singleton births, of which 715,414 singleton neonates delivered at term were included in the final analysis (Figure 1). The characteristics of the multifetal and preterm singleton pregnancies are shown in eTables 1 and eTable 2. There was no noticeable change in the number of neonates enrolled or the prevalence of LBW each year (eFigure 2). This study was approved by the Ethics Committee of Keio University School of Medicine, Tokyo, Japan (No. 20190220, approved November 25, 2019) and the Clinical Research Review Board of the Japan Society of Obstetrics and Gynecology, Tokyo, Japan (No. 2019-15, approved September 14, 2020).

### Measures

Using Japanese standard sex- and parity-specific birthweight percentile curves, a birthweight above the 90th percentile was defined as large for gestational age (LGA), and a birthweight below the 10th percentile was defined as small for gestational age (SGA).^15^ Pre-pregnancy BMI was calculated as a woman’s self-reported pre-pregnancy body weight in kilograms divided by the square of height in meters. The expected GWG at 40 gestational weeks (kg/40 weeks) was computed using a previously reported method.^16^ Using the Institute of Medicine criteria, GWG was classified as inadequate, appropriate, or excessive, based on the pre-pregnancy BMI category. The pre-pregnancy BMI was categorized as underweight (less than 18.5 kg/m^2^), normal weight (18.5 to less than 25.0 kg/m^2^), overweight (25.0 to less than 30.0 kg/m^2^), and obese (30.0 kg/m^2^ or more). The appropriate ranges of GWG based on pre-pregnancy BMI were 12.7–18.1 kg for underweight, 11.3–15.9 kg for normal weight, 6.8–11.3 kg for overweight, and 5.0–9.1 kg for obese patients.^17^ The diagnosis and management of premature labor, premature rupture of membranes, gestational diabetes, HDP, and anemia during pregnancy were performed at the discretion of each obstetrician based on the clinical recommendations of the Japan Society of Obstetrics and Gynecology guideline.^18^ HDP was classified into two categories: hypertension without proteinuria (eg, gestational hypertension [GH], chronic hypertension [CH]) and hypertension with proteinuria (eg, preeclampsia [PE], superimposed preeclampsia [SPE]).^18^ Hypertension was defined as a systolic blood pressure of ≥140 mm Hg and/or a diastolic blood pressure of ≥90 mm Hg, observed on at least two occasions, and proteinuria as the presence of ≥300 mg of protein per day in the urine. Congenital anomalies were defined as those with all types of anomalies registered in the database. These were diagnosed prenatally and/or at the time of delivery by obstetricians and/or after birth by neonatologists. The severity of an anomaly was not recorded.

### Statistical analysis

Data are presented as mean (standard deviation) or number of cases (percentage). Multiple logistic regression analysis was performed to evaluate the relative contributions of various maternal and perinatal factors to LBW among singleton neonates delivered at term. The following independent variables were included in the multivariate model based on prior knowledge,^19^ clinical relevance, and univariable screening: maternal age at delivery, parity (nulliparous or multiparous), method of conception (natural or artificial insemination/in vitro fertilization and embryo transfer [IVF-ET]), pre-pregnancy BMI (underweight/normal/overweight/obese), GWG (inadequate/adequate/excessive), smoking during pregnancy, gestational age at delivery (37 weeks/≥38 weeks), delivery via cesarean section (CS), HDP (none/hypertension without proteinuria/hypertension with proteinuria), anemia during pregnancy, offspring sex, and neonatal congenital anomaly. Multicollinearity was assessed for each independent variable using variance inflation factors, and all values were less than two. Adjusted odds ratios (aORs) and 95% confidence intervals (CIs) were evaluated for the association between LBW and the aforementioned clinical features. Statistical analyses were performed using JMP software (ver. 15; SAS Institute, Cary, NC, USA), and SAS (ver. 9.4; SAS Institute).

## RESULTS

LBW was observed in 18.3% (*n* = 156,450) of the total 852,351 neonates, of which 35.7% (*n* = 55,757) were singleton term pregnancies. The maternal outcomes and the perinatal outcomes of the singleton term neonates are shown in Table 1 and Table 2, respectively. The mothers who delivered LBW neonates were leaner prior to pregnancy and had a lower GWG than those with non-LBW neonates. The proportion of smoking during pregnancy, nulliparity, HDP, delivery at 37 gestational weeks, female offspring, and neonatal congenital anomalies were higher in the LBW group than in the non-LBW group. The proportion of IVF-ET and anemia during pregnancy was lower in the LBW group than in the non-LBW group. Among the LBW infants who were delivered at 37 gestational weeks (*n* = 22,907), 8,426 mothers underwent planned elective CS (36.8%). In the LBW group, 53.5% were classified as SGA, and none were found to be LGA.

**Table 1.** Comparison of maternal characteristics of term singletons based on birthweight status

	LBW (*n* = 55,757)	non-LBW (*n* = 659,657)
Maternal age at delivery, years	32.3 (5.5)	32.3 (5.4)
Maternal age at delivery category		
Teenager	767 (1.4%)	8,207 (1.3%)
35–39	15,009 (26.9%)	178,324 (27.1%)
Over 40	5,253 (9.4%)	60,039 (9.1%)
Maternal pre-pregnancy BMI, kg/m^2^	20.6 (3.4)	21.4 (3.6)
Maternal pre-pregnancy BMI category		
Underweight (BMI < 18.5)	14,188 (25.5%)	107,079 (16.2%)
Normal weight (18.5 ≤ BMI < 25.0)	36,494 (65.5%)	471,857 (71.5%)
Overweight (25.0 ≤ BMI < 30.0)	3,721 (6.7%)	57,453 (8.7%)
Obese (30 ≤ BMI)	1,354 (2.4%)	23,268 (3.5%)
Gestational weight gain, kg/40 w	9.1 (4.4)	10.4 (4.5)
Gestational weight gain category		
Inadequate	40,165 (72.0%)	370,767 (56.2%)
Appropriate	12,798 (23.0%)	222,368 (33.7%)
Excessive	2,794 (5.0%)	66,522 (10.1%)
Smoking during pregnancy	3,300 (5.9%)	25,688 (3.9%)
Nulliparity	34,087 (61.1%)	398,682 (60.4%)
Method of conception: IVF-ET	3,355 (6.0%)	47,934 (7.3%)

BMI, body mass index; IVF-ET, in-vitro fertilization-embryo transfer; LBW, low birthweight.

Data are presented as mean (SD) or *n* (%).

**Table 2.** Comparison of perinatal characteristics of term singletons based on birthweight status

	LBW (*n* = 55,757)	non-LBW (*n* = 659,657)
Gestational weeks at delivery, weeks	37.9 (1.0)	38.9 (1.2)
37 gestational weeks	22,907 (41.1%)	79,962 (12.1%)
Mode of delivery		
Vaginal delivery	32,375 (58.1%)	493,813 (74.9%)
Cesarean section	23,382 (41.9%)	165,844 (25.1%)
Offspring sex, female	33,277 (59.7%)	22,480 (40.3%)
Perinatal complications		
Gestational diabetes	3,195 (5.7%)	38,654 (5.9%)
Hypertensive disorders of pregnancy	5,994 (10.8%)	25,552 (3.9%)
Hypertension only	2,238 (4.0%)	16,776 (2.5%)
Pre-eclampsia	3,756 (6.7%)	8,776 (1.3%)
Anemia during pregnancy	5,342 (9.6%)	95,794 (14.5%)
Birthweight, g	2,365 [2,235–2,442]	3,048 [2,830–3,292]
Neonatal growth category		
Small for gestational age	29,801 (53.4%)	13,846 (2.1%)
Large for gestational age	0	106,696 (16.2%)
Apgar score, 1 minute	8 [8–9]	9 [9–9]
Apgar score, 5 minute	8 [8–9]	9 [9–9]
Neonatal congenital anomaly	2,596 (4.7%)	10,547 (1.6%)

LBW, low birthweight.

Data are presented as mean (SD), median [IQR, 25th–75th percentile], or *n* (%).

Table 3 shows the associations between different clinical factors and LBW in singleton term infants. Compared to women aged 25–34 years, those aged <25 years had a higher risk of delivering LBW infants. After adjustment for other risk factors, the OR was 1.21 (95% CI, 1.11–1.31) for those younger than 20 years old and 1.20 (1.16–1.24) for those aged 20–24 years. On the other hand, women aged 35–39 years and 40 years or older both had a 5–8% lower risk of having LBW when adjusted for other risk factors.

**Table 3.** Associations of maternal and perinatal factors with low birthweight in term singletons

Category		Unadjusted OR (95% CI)		Adjusted OR (95% CI)	
Maternal age at delivery	25–34 years	ref		ref	
	<20 years	1.12	(1.04–1.21)	1.21	(1.11–1.31)
	20–24 years	1.10	(1.06–1.14)	1.20	(1.16–1.24)
	35–39 years	1.01	(0.99–1.03)	0.94	(0.92–0.96)
	≥40 years	1.05	(1.02–1.08)	0.92	(0.89–0.95)
Parity	Multiparous	ref		ref	
	Nulliparous	1.03	(1.01–1.05)	1.23	(1.20–1.25)
Method of contraception	Spontaneous or AIH	ref		ref	
	IVF-ET	0.82	(0.79–0.85)	0.66	(0.63–0.69)
Pre-pregnancy BMI	Normal	ref		ref	
	Underweight	1.71	(1.68–1.75)	1.64	(1.61–1.68)
	Overweight	0.84	(0.81–0.87)	0.82	(0.79–0.85)
	Obese	0.75	(0.71–0.80)	0.56	(0.53–0.60)
Gestational weight gain	Appropriate	ref		ref	
	Inadequate	1.88	(1.84–1.92)	1.92	(1.88–1.96)
	Excessive	0.73	(0.70–0.76)	0.65	(0.62–0.68)
Gestational weeks at delivery	≥38 weeks	ref		ref	
	37 weeks	5.06	(4.96–5.15)	4.53	(4.44–4.62)
Mode of delivery	Vaginal delivery	ref		ref	
	Cesarean section	2.15	(2.11–2.19)	1.56	(1.53–1.59)
Hypertensive disorders of pregnancy	None	ref		ref	
	Hypertension only	1.70	(1.63–1.78)	1.83	(1.75–1.92)
	Preeclampsia	5.46	(5.25–5.67)	5.62	(5.38–5.87)
Smoking during pregnancy		1.55	(1.50–1.61)	1.78	(1.71–1.85)
Anemia during pregnancy		0.62	(0.61–0.64)	0.60	(0.58–0.62)
Female neonate		1.59	(1.56–1.62)	1.70	(1.67–1.73)
Neonatal congenital anomaly		3.01	(2.88–3.14)	2.86	(2.73–3.00)

AIH, artificial in insemination with husband’s semen; BMI, body mass index; CI, confidence interval; HDP, hypertensive disorder of pregnancy; IVF-ET, in-vitro fertilization-embryo transfer; OR, odds ratio.

Pre-pregnancy underweight and inadequate GWG also contributed to LBW independently; the risk of LBW for being underweight prior to pregnancy was 1.64 (95% CI, 1.61–1.68) compared to normal pre-pregnancy weight, while it was 1.92 (95% CI, 1.88–1.96) for those with inadequate GWG compared with those with appropriate GWG. Nulliparity (aOR 1.23; 95% CI, 1.20–1.25), smoking during pregnancy (aOR 1.78; 95% CI, 1.71–1.85), delivery at 37 gestational weeks (aOR 4.53; 95% CI, 4.44–4.62), CS (aOR 1.56; 95% CI, 1.53–1.59), female offspring (aOR 1.68; 95% CI, 1.65–1.72), and congenital anomalies (aOR 2.86; 95% CI, 2.72–2.99) also independently contributed to the risk of LBW in term singletons. Compared to those with no HDP, those with hypertension without proteinuria (eg, GH and CH) had an increased risk of delivering LBW neonates (aOR 1.83; 95% CI, 1.75–1.92), whereas hypertension with proteinuria (eg, PE and SPE) was associated with an even higher risk of LBW in term singletons (aOR 5.62; 95% CI, 5.38–5.87).

## DISCUSSION

Using the pregnancy birth registry data, this study revealed that more than one-third (35.7%) of the nation’s LBWs were singleton term births. Multiple factors were identified to be independently associated with LBW singleton infants born at term. The associated maternal factors included age, parity, mode of conception, pre-pregnancy BMI, GWG, and smoking during pregnancy. Associated perinatal factors included HDP, anemia during pregnancy, mode and timing of delivery, and the neonatal factors included offspring sex and presence of a congenital anomaly. Our data are among the first to provide evidence that LBW is not only an issue with multiples and preterm birth but also with term birth. As such, LBW term infants and their associated factors should also be addressed when discussing preventive strategies.

Maternal underweight and inadequate GWG remained independent risk factors for LBW in term singletons after adjusting for other traditionally reported factors, which was consistent with previous findings explored in the context of preterm birth.^20^^,^^21^ Underweight and low GWG has been a problem among Japanese women in recent years. According to the Japanese National Health and Nutrition Survey, 19.8% of Japanese women in their 20s were underweight, higher than that in other developed countries, and the GWG tended to be lower in Japanese mothers than in non-Hispanic white mothers.^22^ As maternal body image and eating habits during pregnancy are modifiable, raising awareness and educating women on these issues may reduce the risk of LBW. It should be noted that Asian women are known to have reduced beta-cell function and are more likely to develop glucose intolerance than Caucasians or Hispanics, even if insulin resistance increases similarly during pregnancy.^23^^,^^24^ Therefore, setting appropriate goals for pre-pregnancy BMI and GWG in Asian mothers may require further study.

The association between advanced maternal age and LBW has been inconsistent, depending on the study population and point in time.^25^ Increased maternal age is often associated with other risk factors for LBW, such as the mode of conception and obstetric complications. We adjusted for possible confounding factors through logistic regression analysis and found a negative association between advanced maternal age and LBW in term singletons, irrespective of the mode of conception and obstetric complications.

LBW and its associations with IVF-ET, the presence of anemia during pregnancy, and delivery with CS have not been well investigated. In the present study, a negative association was observed between IVF-ET and LBW. This observation is supported by one finding that neonates conceived by frozen-thawed embryo transfer are heavier than those conceived through natural conception or fresh embryo transfer.^26^ Although the detailed method of IVF-ET was missing, conception by IVF-ET may have reduced the risk of LBW, given that the mainstream method in Japan is frozen-thawed embryo transfer. The mechanism underlying anemia as a protective factor against LBW in our study is unclear. Since many pregnant women diagnosed with anemia often receive supplemented iron in Japan, it is possible that iron administration had a positive effect on the reduction of LBW, rather than anemia itself.^27^ Finally, delivery by CS was independently associated with LBW in term neonates. In the present study, 36.8% of LBW term infants delivered at 37 weeks of gestation were done so via elective CS. Due to the lack of detailed indications for elective CS, it is difficult to make concrete assumptions. However, if nonmedically indicated deliveries are included at a fixed number, scheduling them at later weeks might lead to a reduced risk of LBW in CS births.

It has been reported that maternal body composition affects fetal growth differently, depending on the offspring sex.^28^ The present study indicated that infant sex is associated with birthweight, with female neonates having a higher risk of LBW than male neonates among term singletons. Sex differences have also been reported in the risk of future metabolic syndrome in a prospective cohort of infants with very LBW (<1,500 g), where the risks were higher in men than in females.^29^ There is indeed evidence that sex-specific differences exist in the association between birthweight and adult resting metabolic rate, a factor associated with weight gain and metabolic syndrome.^30^ Given the differences in the risk of future health disorders, modifying the cut-off points for LBW based on sex may be relevant.

In the present study, neonatal congenital anomalies were associated with a higher risk of LBW in term singletons. Although not preventable, neonates with congenital anomalies may have neuropsychiatric and endocrine disorders, which may intervene in the association between LBW and the risk of developing NCD in the future. Previous studies have rarely explored this issue and have either excluded neonates with congenital anomalies or analyzed them together with neonates without anomalies. Examining LBW and the risk of developing NCDs in children with congenital anomalies may provide new insights into their health care.

The present study has several limitations. First, Japan may have different frequencies of preterm birth or LBW and their subgroups than other developed countries (eFigure 1). As such, generalizing the results to other countries or regions requires caution. Second, the study participants may not be representative of the entire population. The prevalence of LBW in Japan was reported to be 9.4% in the vital statistics of Japan, whereas it was 18.3% in our database. This difference is likely because Japanese national perinatal database consists mainly of secondary and tertiary hospitals. According to the Japanese vital statistics, approximately 470,000 LBW neonates were born in Japan during the study period, but only 156,450 LBW neonates were included in the study after excluding those with missing data. Therefore, it is estimated that our study involved more than one-third of the total LBW neonates in the country, and we considered it appropriate to investigate the association of LBW within the nation using these data. Third, because this database did not allow data querying, the exclusion of many cases with unknown details could not be avoided (36%). Details of medical information were not recorded in the database, which restricted further discussion on factors associated with LBW. An example would be a case of elective CS for breech presentation performed at 37 gestational weeks. If a detailed explanation of the timing of the surgery was known, such as whether other maternal complications existed or whether it was performed at the patient’s request, further discussions could have been possible. Furthermore, the database did not contain information on the socioeconomic status of the patients as well as environmental and behavior risks, such as maternal work and psychological stress, consumption of alcohol and caffeine, and environmental exposures; therefore, impact of the unmeasured variables on the results could not be assessed. Fourth, the broad definition of congenital anomalies in this study might have allowed cases that were not directly associated with LBW, such as non-syndromic orofacial clefts or clinically non-significant heart defects, such as a small ventricular septal defect. However, the positive association between LBW and congenital anomalies based on this definition raises the possibility that some children with congenital anomalies are at a higher risk of LBW and, thus, are at a higher risk for NCD, requiring careful follow-up. Lastly, repeated entries were allowed for one woman with more than one pregnancy; statistical methods, such as a multi-level model, might have been more appropriate. The database lacked a linkage between the identification number of the mother and the child, making such a statistical analysis difficult. Nevertheless, there have been few reports on the factors associated with LBW in term births on such a large scale, and we consider that our results would be valuable in understanding LBW and its associated factors in this population and in discussing effective strategies to reduce it.

In conclusion, using the Japanese pregnancy birth registry data, which included 715,414 singleton neonates delivered at term from an Asian population, this study showed that LBW comprised not only preterm and multiple births but also term singleton births in comparable proportions. This study expounded on previous findings by showing that factors known to elevate the risk of LBW in preterm births, such as smoking, maternal malnutrition, and presence of HDP, were also independently associated with LBW in term births. Our study provided data on the prevalence of LBW, which has not been well addressed in the past, enabling us to evaluate the status and monitor progress within and across countries on this issue. Although the situation may be different in each country, our results suggest that strategies to reduce preterm births and LBW could be equally effective in preventing LBW of term births. Therefore, continued and accelerated efforts are expected to contribute to the overall reduction of LBW worldwide.

## SUPPLEMENTARY MATERIAL

eTable 1. Neonatal characteristics in multiple pregnancy

eTable 2. Maternal and perinatal characteristics in singleton pregnancy with preterm birth (n = 82,871)

eFigure 1. Comparisons of preterm birth and low birthweight in 11 countries.^4^^,^^9^^–^^13^

eFigure 2. Changes in number of births (bar graph) and prevalence of low birthweight (line graph), 2013–2017
