# Supplementary material for: The Associated Factors of Low Birthweight Among Term Singletons in Japan: A Pregnancy Birth Registry Analysis
Source: J Epidemiol. 2023 Sep 5;33(9):450–5. doi: 10.2188/jea.JE20210483 (PMC10409524; doi:10.2188/jea.JE20210483)
Supplement: Supplementary file 1 [file je-33-450-s001.pdf]

eTable 1. Neonatal characteristics in multiple pregnancy

Neonatal characteristics (n=54,127)	n	(%)
Twin pregnancy	52,832	(97.6%)
Monochorionic-monoamniotic twin	303	(0.6%)
Monochorionic-diamniotic twin	19,092	(35.3%)
Dichorionic-diamniotic twin	32,937	(60.9%)
Unknown	500	(0.9%)
Triplet pregnancy	1,250	(2.3%)
Quadruplet pregnancy	45	(0.08%)
Preterm birth, <37 gestational weeks	28,424	(52.5%)
Offspring sex, female[a]	26,978	(49.8%)
Low birthweight, <2,500 g	39,284	(72.6%)
Twin-twin transfusion syndrome	1,558	(2.9%)

[a]Data were missing in 19 pregnancies.

eTable 2. Maternal and perinatal characteristics in singleton pregnancy with preterm birth (n=82,871)

Characteristics	Mean (SD) or n (%)
Maternal age at delivery, years	32.5 (5.5)
Teenager	1,142 (1.4%)
35–39 year	23,583 (28.5%)
40 years or older	8,028 (9.7%)
Maternal pre-pregnancy BMI, kg/m^2	21.4 (3.9)
Underweight (BMI <18.5)	15,996 (19.3%)
Normal weight (18.5≤BMI<25.0)	55,348 (66.8%)
Overweight (25.0≤BMI<30.0)	8,079 (9.7%)
Obesity (BMI ≥30)	3,448 (4.2%)
Smoking during pregnancy	2,330 (2.8%)
Nulliparity	39,931 (48.2%)
Method of conception	
Spontaneous conception	71,805 (86.6%)
Ovulation	1,822 (2.2%)
IUI	1,554 (1.9%)
IVF-ET	4,904 (5.9%)
ICSI	1,312 (1.6%)
Unknown	1,474 (1.8%)
Gestational weeks at delivery, weeks	33.6 (3.1)
Mode of delivery	
Vaginal delivery	33,149 (40.0%)
Vacuum delivery	2,401 (2.9%)
Forceps delivery	352 (0.4%)
Elective cesarean section	8,268 (10.0%)
Emergency cesarean section	38,268 (46.2%)
Unknown	433 (0.5%)

BMI, body mass index; HELLP, Hemolysis, Elevated Liver enzymes, and Low Platelet count; IUI, intrauterine insemination; IVF-ET, *in-vitro* fertilization-embryo transfer; ICSI, intracytoplasmic sperm injection; LEEP, loop electrosurgical excision procedure; PROM, premature rupture of membrane.

[a]Data were missing in 59 pregnancies.

eTable 2. (continued)

Characteristics	Mean (SD) or n (%)
Perinatal complications	
Cervical insufficiency	2,360 (2.8%)
Anemia during pregnancy	7,271 (8.8%)
Gestational diabetes	4,820 (5.8%)
Preterm PROM	24,768 (29.9%)
Hypertensive disorder in pregnancy	13,679 (16.5%)
Preeclampsia	9,757 (11.8%)
Fetal growth restriction	10,035 (12.1%)
HELLP syndrome	942 (1.1%)
Placental abruption	3,593 (4.3%)
Placental previa or low-lying placenta	6,517 (7.9%)
Past medical history or comorbidities	
Prior conization or LEEP	1,011 (1.2%)
Offspring sex (female)[a]	36,082 (43.5%)
Birthweight, g	2,055 (641)
Low birthweight	61,461 (74.2%)
Small for gestational age	24,258 (29.3%)
Large for gestational age	22,388 (27.0%)
Neonatal congenital anomaly	2,367 (2.9%)

BMI, body mass index; HELLP, Hemolysis, Elevated Liver enzymes, and Low Platelet count; IUI, intrauterine insemination; IVF-ET, *in-vitro* fertilization-embryo transfer; ICSI, intracytoplasmic sperm injection; LEEP, loop electrosurgical excision procedure; PROM, premature rupture of membrane.

[a]Data were missing in 59 pregnancies.

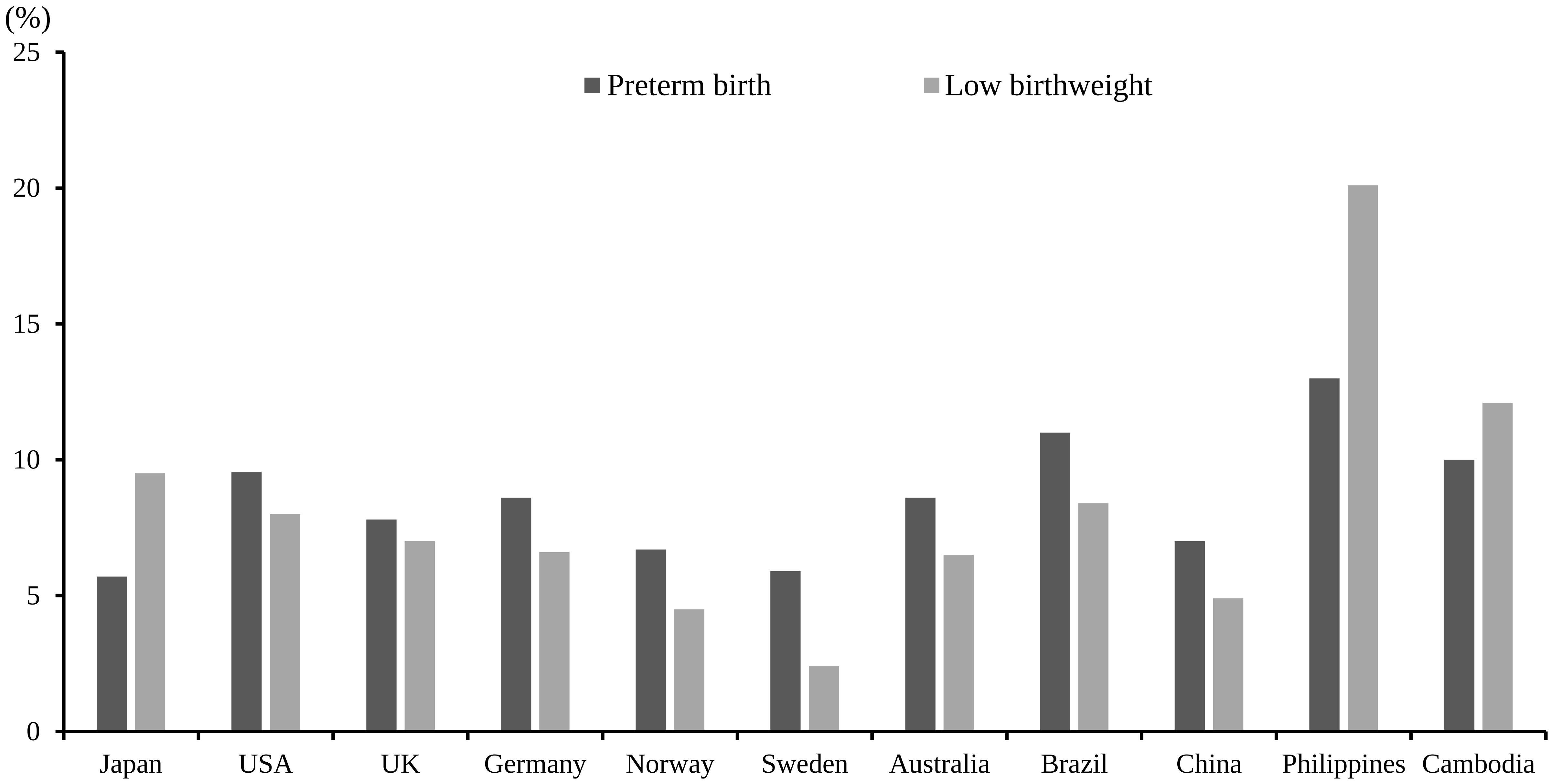

eFigure 1. Comparisons of preterm birth and low birthweight in 11 countries [4, 9-13]

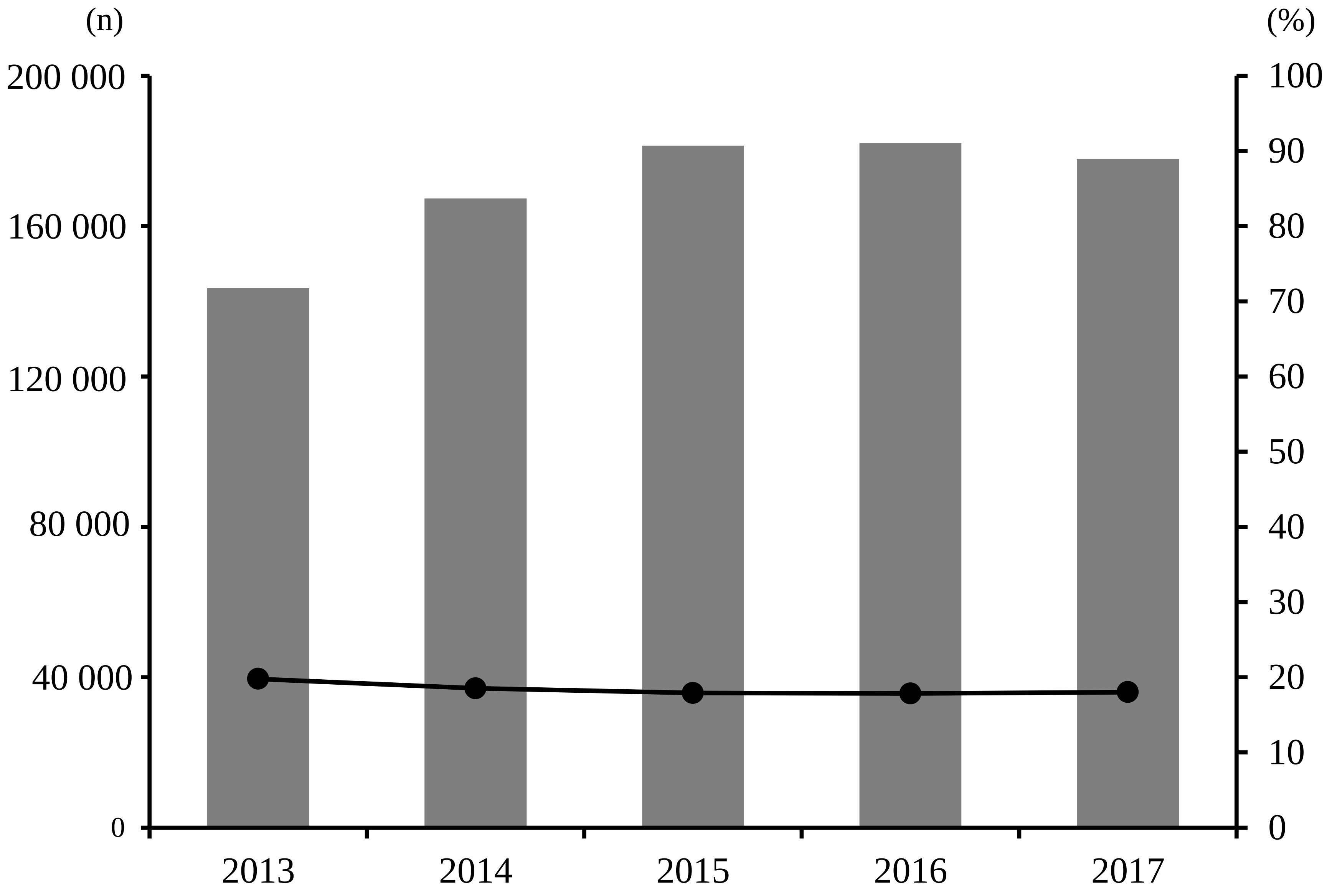

eFigure 2. Changes in number of births (bar graph) and prevalence of low birthweight (line graph), 2013–2017
